# G × E interactions as a basis for toxicological uncertainty

**DOI:** 10.1007/s00204-023-03500-9

**Published:** 2023-06-01

**Authors:** Ilinca Suciu, David Pamies, Roberta Peruzzo, Petra H. Wirtz, Lena Smirnova, Giorgia Pallocca, Christof Hauck, Mark T. D. Cronin, Jan G. Hengstler, Thomas Brunner, Thomas Hartung, Ivano Amelio, Marcel Leist

**Affiliations:** 1 In Vitro Toxicology and Biomedicine, Department Inaugurated By the Doerenkamp-Zbinden Foundation, University of Konstanz, Universitaetsstr. 10, 78457 Constance, Germany; 2 Department of Biological Sciences, University of Lausanne, 1005 Lausanne, Switzerland; 3 Department of Molecular and Cell Biology, University of California, Berkeley, CA 94720 USA; 4 Centre for the Advanced Study of Collective Behaviour, University of Konstanz, 78457 Constance, Germany; 5 Center for Alternatives to Animal Testing (CAAT), Johns Hopkins University, Bloomberg School of Public Health, Baltimore, MD 21205 USA; 6 CAAT Europe, University of Konstanz, 78457 Constance, Germany; 7 Department of Cell Biology, University of Konstanz, 78457 Constance, Germany; 8 School of Pharmacy and Biomolecular Sciences, Liverpool John Moores University, Byrom Street, Liverpool, L3 3AF UK; 9 Leibniz Research Centre for Working Environment and Human Factors, Technical University Dortmund, 44139 Dortmund, Germany; 10 Biochemical Pharmacology, Department of Biology, University of Konstanz, 78457 Constance, Germany; 11 Division for Systems Toxicology, Department of Biology, University of Konstanz, 78457 Constance, Germany; 12 Biological Work and Health Psychology, Department of Psychology, University of Konstanz, 78457 Constance, Germany

**Keywords:** Epigenetics, Model system, Safety factor, Toxicokinetics, Resilience, AOP

## Abstract

To transfer toxicological findings from model systems, e.g. animals, to humans, standardized safety factors are applied to account for intra-species and inter-species variabilities. An alternative approach would be to measure and model the actual compound-specific uncertainties. This biological concept assumes that all observed toxicities depend not only on the exposure situation (environment = E), but also on the genetic (G) background of the model (G** × **E). As a quantitative discipline, toxicology needs to move beyond merely qualitative G** × **E concepts. Research programs are required that determine the major biological variabilities affecting toxicity and categorize their relative weights and contributions. In a complementary approach, detailed case studies need to explore the role of genetic backgrounds in the adverse effects of defined chemicals. In addition, current understanding of the selection and propagation of adverse outcome pathways (AOP) in different biological environments is very limited. To improve understanding, a particular focus is required on modulatory and counter-regulatory steps. For quantitative approaches to address uncertainties, the concept of “genetic” influence needs a more precise definition. What is usually meant by this term in the context of G** × **E are the protein functions encoded by the genes. Besides the gene sequence, the regulation of the gene expression and function should also be accounted for. The widened concept of past and present “gene expression” influences is summarized here as G_*e*_. Also, the concept of “environment” needs some re-consideration in situations where exposure timing (E_*t*_) is pivotal: prolonged or repeated exposure to the insult (chemical, physical, life style) affects G_*e*_. This implies that it changes the model system. The interaction of G_*e*_ with E_*t*_ might be denoted as G_*e*_** × **E_*t*_. We provide here general explanations and specific examples for this concept and show how it could be applied in the context of New Approach Methodologies (NAM).

## Uncertainty

Toxicological data are always associated with uncertainty. This uncertainty needs to be considered when the primary test data are converted to legally binding exposure limits, or thresholds for safe exposure. A typical approach to account for uncertainty in exposure thresholds is to reduce the experimental threshold dose by a safety factor (SF, also called uncertainty factor). This is usually accomplished by dividing the experimental threshold by a SF of 100, to arrive at the regulatory value that is suitable for application for human exposure. The SF may be larger if particularly susceptible populations need to be protected, or if the knowledge base is weak. In standard cases (with a robust and refined knowledge base), a factor of 10 is considered to account for human (inter-individual) variability. Another factor of 10 is applied to buffer errors due to extrapolation from a model system (animal) to man (inter-species conversion) (Chapman et al. 1998; Dankovic et al. 2015; Dorne and Renwick 2005). This standardized approach follows the precautionary principle, and is relatively easy to apply without a need for additional scientific information on given chemicals. However, if applied in a stereotypic (non-flexible) way, this approach may under- or over-estimate the uncertainty of risk of individual substances. To make risk assessment more scientifically justifiable, an alternative approach would be to better understand and quantify all factors that contribute to uncertainty, and then to study or model/predict their role for given chemicals under evaluation.

Although the above considerations are generally applicable, each field, industrial sector or national legislation has developed slightly different procedures to relate the primary testing data, usually expressed as benchmark doses (BMD) or as no observed adverse effect levels (NOAEL), to exposure limits for human populations. Depending on the legislation, such legally binding exposure limits may then be called reference doses (RfD), acceptable daily intake (ADI), tolerable daily intakes (TDI) or maximal workspace concentrations (MAC).

## Main types of uncertainties considered

Many studies show that previously and currently used animal models have many levels of uncertainty with respect to human prediction (Grass and Sinko 2002; Luechtefeld et al. 2018; Ly Pham et al. 2020; Natsch et al. 2023; Smirnova et al. 2018; Wang and Gray 2015). In the future, New Approach Methodologies (NAMs) and the strategies to use and combine them will play important roles (Krebs et al. 2020; Leist et al. 2014; Mone et al. 2020; Pallocca and Leist 2022). The uncertainties of such modern model systems require more research, and this needs to cover all variabilities covered by classical safety factors. In general, classical toxicological SFs are considered to contain a toxicodynamic and a toxicokinetic component. For instance, the inter-individual factor of 10 comprises a 10^0.5^ (square root of 10 ≈ 3.2) component for toxicokinetic variation and a similarly sized component reflecting toxicodynamic uncertainty. Similarly, the inter-species factor is (in some legislations) considered to consist of a factor of 4 for toxicokinetic differences and an assumed factor of 2.5 for the difference in toxicodynamics between animals and humans. In the case of NAMs, it is possible that novel strategies will be required to not only determine hazard and basic absorption-distribution-metabolism-excretion (ADME) properties (Tsaioun et al. 2016), but also to determine their uncertainty relative to the human population.

Major components of uncertainty in the setting of exposure-based limits are differences: (1) within susceptible and less susceptible human subpopulations, (2) between tissues and cells that may be affected, (3) of life stages (e.g. foetal vs adult), and (4) of past exposures/disease states. In addition, a further set of uncertainties comes from the relation of test methods (and the model system used for them) to the target population, i.e. (5) cross-species differences in case of animal models, or (6) those associated with various test method protocols of NAM, even when the insult is the same. In a widened concept, going beyond toxicology, and embracing issues of general health and ageing, the overall life-style (for humans) comes in as another important component (beneficial or negative modifier function) for cumulative risk.

All considerations on toxicological uncertainty are based on five major drivers:

*Xenobiotic metabolism* A large fraction of xenobiotics are metabolized, which usually shortens the periods during which the parent compounds can interact with organs and tissues. Besides the host’s cells, such as hepatocytes, the microbiome may also contribute to metabolism. Different metabolic rates can affect maximum plasma concentrations (*C*_max_) and also the concentration–time courses in target tissues (Hassan et al. 2022; Oesch et al. 2022; Ren et al. 2022). In some cases, metabolism can lead to the generation of intermediates with increased toxicity, such as reactive electrophiles, and the relative speed of various metabolic pathways can determine the concentration-, time-courses and location of such toxicants generated in the body (Capinha et al. 2021, 2023). Thus, metabolism often contributes a major fraction of inter-species differences, inter-individual human differences, and target cell specificities. Many individual NAMs are still missing this component (Coecke et al. 2006).

*Distribution* (comprising absorption and elimination): many toxicants (or their metabolites) are substrates of transporters. This condition determines their localization, accumulation, or elimination from body compartments (Grass and Sinko 2002; Schildknecht et al. 2015). The distribution of transporters and their enzymatic properties concerning xenobiotics (*K*_m_, *V*_max_) can thus affect tissue concentrations of chemicals and, thereby, their toxicologically effective concentrations at target sites. In many cases, target organ specificities are driven by distribution phenomena. For weak bases and acids, the distribution is often determined by transporters, and by the pH differences of body compartments.

*Need/Demand* For toxicants that impair certain cellular functions, the threshold of toxicity is determined by the extent the cell depends on these functions (or can buffer functional losses). For instance, carbon monoxide poisoning leads to a lack of oxygen supply and thus to an impaired respiration in the body. However, some cells depend more on respiratory ATP generation than others, and this characteristic determines the target organ specificity. Similarly, toxicants may affect organ development only during a specific time window when organogenesis critically depends on the respective target pathway (Balmer et al. 2014; Dreser et al. 2020; Meisig et al. 2020; Schildknecht et al. 2017).

*Resilience* Cells and tissues have evolved extensive and elaborate mechanisms to compensate, buffer and repair damage (Smirnova et al. 2015). As such resilience requires time to build up and often fades later, this is an example of the importance of insult timing. For example, hepatic levels of CYP2E1 strongly decrease during repeated exposure to cytotoxic doses of compounds that are metabolically toxified by this enzyme, such as CCl_4_ (Ghallab et al. 2019b). Moreover, export carriers of hepatocytes can be upregulated to protect the cells from cytotoxicity due to overloading with bile acids or xenobiotics (Ghallab et al. 2019a). The use of stress response pathways differs largely, so that a given insult may be innocuous for one cell type, but detrimental for another (Gutbier et al. 2018b; Harris et al. 2018; Smirnova et al. 2015; Wijaya et al. 2022). According to the toxicological concept of the adverse outcome pathway (AOP) (Leist et al. 2017), it is possible to include resilience factors as a “modulatory influence” on key events and within the description of key event relationships. For most AOPs, very little (or no) quantitative information is available on these issues (Spinu et al. 2020).

*Target biochemistry/AOP* Last, but not least, biochemical targets of toxicants may play a role. Within the AOP concept, this would mean that triggering of molecular initiating events (MIE) would be different: if the expression levels, post-translational modifications or localization of a toxicant target are changed, then the affinity and extent of the MIE would be altered. This could lead to altered pathways of toxicity on the molecular/cellular level (Kleensang et al. 2014) and an enhanced, or attenuated, triggering of the AOP. In addition, such biochemical changes may lead to the triggering of AOPs otherwise not triggered. In more general terms, the patterns of activation of AOP networks may be altered.

**Fig. 1:**
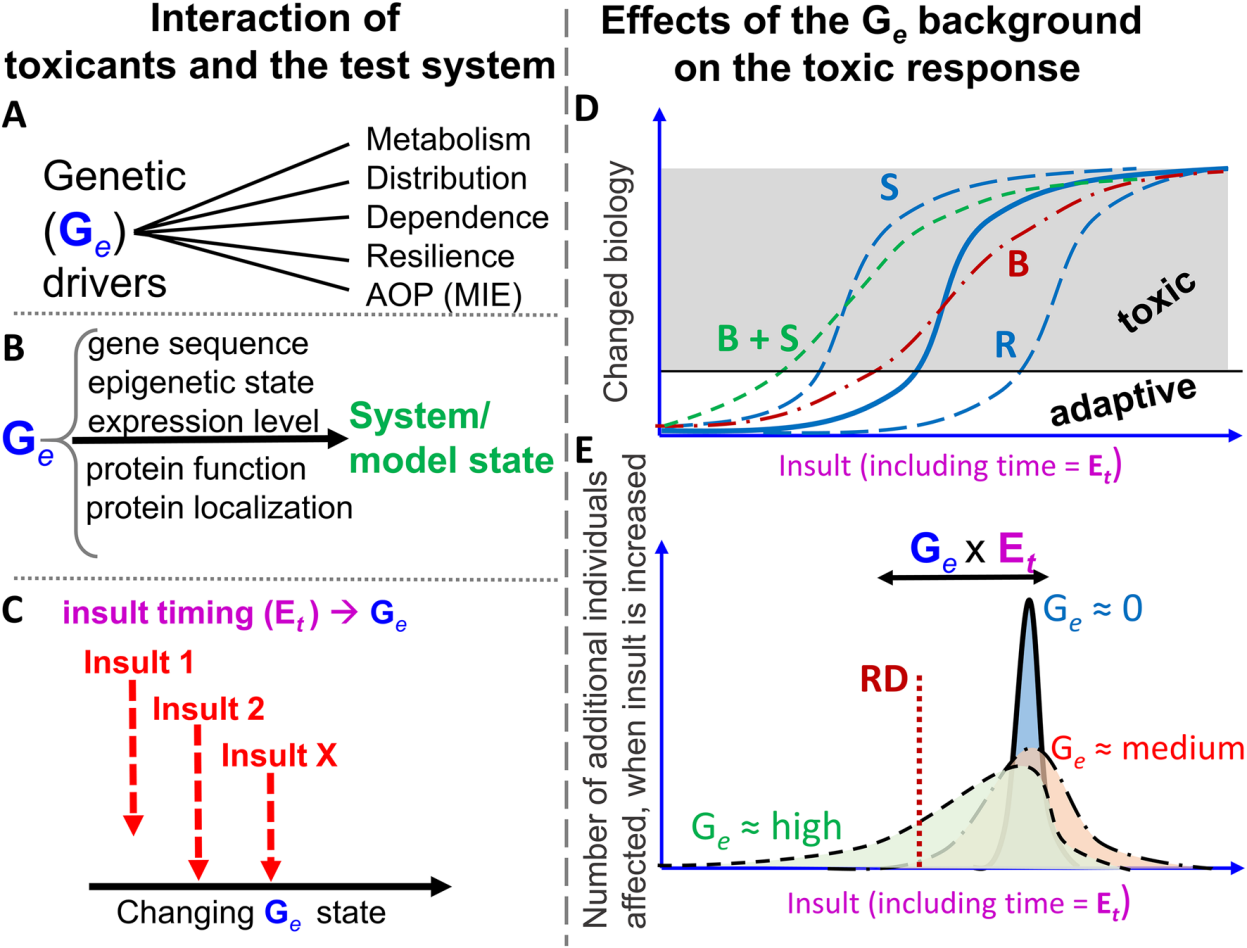
Relationship of insult, G_e_ and toxicity outcomes. Several aspects of mutual interactions are displayed. The left side (subfigures **A**–**C**) deals with interactions of toxicants and test system (toxicants are considered here in a very wide sense, comprising all adverse influences, such as chemicals, radiation, infectious agents and other stressors). The right side (subfigures **D**–**E**) deals with effects of the G_*e*_ background on the toxic response. **A** The genetic component (G_*e*_ state) can affect toxicity in many ways. Five major drivers are shown and further explained in the main text. MIE refers to molecular initiating events (MIE of adverse outcome pathways (AOP)). **B** The genetic component in the classical G × E concept is not adequately described by “primary genetic sequence”. In reality, it is not the nucleic acid component of a gene that determines toxic responses, but the function of the protein that is encoded by the gene. The “real” meaning of “G” includes therefore at least the factors: gene sequence, epigenetic state, expression level, and post-translational processing. Altogether, this G_*e*_ state determines the properties of the model system. **C** The environment (E) component of the classical G × E concept is composed of chemical, physical and life style factors. It is sometimes considered independent of (orthogonal to) genetic (G_*e*_) influences. This is an oversimplification in situations of prolonged or repeated insult. Under such conditions, it needs to be considered that insults not only contribute to toxicity as an endpoint, but that they also alter the test system. This involves in many cases a change of G_*e*_. Thus, a system with a given initial G_*e*_ state during the first insult may have a different G_*e*_ state upon a secondary or later insult, because the first insult changed the system. The timing of exposures to toxicants or other insults (E_*t*_) needs consideration for a quantitative strategy to predict the extent and variability of toxicity. **D** The solid (blue) line shows a typical biological response to an insult (assumed to be the average within a test population): with an increasing insult, the biological system changes. It is assumed that up to a certain level, the changes are within a normal homeostatic regulation range and can be called adaptive; beyond this threshold (grey area) the changes are classified as toxicity. The dashed curves exemplify responses in particularly sensitive (S) or resistant (R) individuals. The dash-dotted line (B, red) shows a broadened response (i.e. it refers to a population with more variation). The short-dashed line (B + S, green) shows a broadened response in a particularly sensitive subpopulation. While the insult is clearly defined by the values on the *x*-axis, the resultant biological deviation differs between members of the population. The sensitivity differences of its members are assumed to be due to genetic variation. The toxicity threshold is therefore reached at different insult intensities for individuals with different genetic background. **E** There is always an uncertainty of binary classifications (toxic vs non-toxic), which is largest at insult levels corresponding to the toxicity threshold. This can be visualized by frequency distribution curves showing how many additional individuals would be classified as affected (toxicity onset) at the next incremental insult step. If there is no (or very low) genetic variability (G_*e*_ = 0, solid, blue), the uncertainty distribution is narrow. A moderate genetic variability (dash-doted, red) leads to broadening of the distribution (some individuals affected at clearly lower toxicant levels, some at clearly higher levels). A high genetic variability (dashed, green) leads to further broadening, and may lead to asymmetric shapes, e.g. with a particularly sensitive sub-population. Given a certain reference dose (RD, e.g. accepted daily intake), the G_*e*_ = 0 population would be safe. In the red (dash-dotted) population, some individuals would be endangered. Many individuals in the green (dashed) population would be victims of toxicity. Thus, the interaction of genetic factors (G_*e*_) and insult (including its timing, E_*t*_), i.e. G_*e*_ × E_*t*_, determines response variability and affects setting of safe reference doses

All the above drivers of variation have genetic components (Fig. 1A). For instance, a mutation in a gene may alter its affinity for a toxicant, or the increased expression level of a protein may lead to some attenuation of toxicity. Therefore, it appears essential to consider the effect of toxicants in light of given “genetic backgrounds”.

## G × E concept

The G** × **E concept dates back nearly 100 years and the notion of an interaction of heritable factors (genetic setup) and non-heritable influences (environment, lifestyle) on disease outcome and on different life performance parameters is well-established in many scientific fields (bit.ly/3YEbuuc). There is agreement that both genetics (nature, G) and the environment (“nurture” or external insults, E) together (G** × **E) affect pathological processes. However, the concept is rarely used in a fully quantitative way. Exact definition of what is meant by G, E, or the connector (**×**), are rarely given. They are mostly qualitative descriptions and they may vary greatly between different studies.

A critical aspect of the G** × **E discussion is the underlying models and causes. While it has been postulated that the interaction (G** × **E) of genes (G) and the environment (E) is only of statistical nature (no biological significance), it is now largely accepted that there are defined underlying biological mechanisms and that the two components can influence each other. Advanced examples of this concept are available from the field of pharmacogenetics (effect of drug dose and genetic background on maximum drug concentrations). One example refers to organophosphate toxicity: paraoxonase polymorphisms and toxicant dose interact to determine acetylcholine esterase inactivation or overall toxicity, e.g. for sarin (Bolt 2023).

While historical models consider the role of genes as binary input (on/off), it is increasingly evident that G-effects can be polygenic and that expression of genes can vary on a continuous scale. An extreme example of G** × **E pathology is the autosomal recessive disorder phenylketonuria. It is caused by a rare gene mutation (disease frequency 1:15,000) that leads to defects in brain development followed by intellectual disability. However, if the biallelic mutation is identified at birth, and a diet with low levels of the amino acid phenylalanine is applied, no disease phenotype will occur and development occurs normally. Also, the expression of many polygenetic disease syndromes can be largely affected by diet, e.g. coronary artery disease, type 2 diabetes, and non-alcoholic fatty liver disease. For instance, there is an extensive directory of G** × **E interactions relevant to cardiometabolic traits and ensuing disease (Parnell et al. 2014). In many cases, a gene may have dozens or even hundreds of variants, alleles and mutated forms, in addition to copy number variations and changes in enhancer regions. This can lead to a broad range of activities of the encoded protein. One example is the melanocortin-4 receptor (MC4R). Its mutations and loss are associated with obesity. Many partially active mutations allow the drug setmelanotide (a MC4R agonist) to be clinically beneficial (Abbasi 2021; Fatima et al. 2022). Another clinically relevant example is the cystic-fibrosis-related chloride transporter (CFTR). Mutations can be associated with a range of residual activities, and drug effects depend on the type of mutation (Fajac and Girodon 2020).

While the acceptance of the G** × **E concept, i.e. mutual interaction of genes and environment, has provided a unifying solution for the nurture vs. nature debate, many additional steps are required to sharpen the concept and allow eventually a quantitative application e.g. to the definition of uncertainty in risk assessment.

## G × E examples in toxicology and beyond

The examples of G** × **E interactions in pharmacology and toxicology are legion, and only few outstanding examples will be given. For instance, allelic variants for drug acetylation or hydroxylation by cytochrome P450-2D6 have a major influence on their metabolic capacity and therewith on the concentrations of their substrates. Mutations of the tumour suppressor p53 can affect the toxicity of irradiation, chemotherapeutics or genetic modifiers (Levine and Oren 2009; Panatta et al. 2022a, b; Sabapathy and Lane 2018). The concept of G** × **E for metal toxicity is also very broadly explored (Broberg and Pawlas 2022). A more focussed example is Fava bean consumption, which results in haemolytic anaemia only in “normal” subjects, but not when the *G6PD* gene product is deficient. An example for a functional polymorphism comes from the antioxidant gene *NQO1* where a single small change in the coding sequence (rs1800566) favours NOx-induced lung injury (Basharat et al. 2016).

Species-dependent susceptibilities to potent toxins also provide a clear illustration of the genetic component. Organisms using tetrodotoxin (TTX) for defence have altered the genetic sequence of their own potential toxin target, a sodium channel, so that the protein does not bind TTX (Geffeney et al. 2019). Moreover, snakes using α-neurotoxins are resistant because of mutations in their nicotinic receptors (the usual target) that prevent toxin binding (Khan et al. 2020).

Similar resistance versus susceptibility phenomena are well studied in the case of infectious diseases, where the presence or absence of a suitable receptor determines the host range of viruses or the disease risk for individuals (Liu et al. 1996; V'kovski et al. 2021). The more than 400 identified inborn-errors-of-immunity provide numerous examples of specific human genotypes linked to an often narrow spectrum of infectious diseases (Casanova and Abel 2022; Notarangelo et al. 2020). A case in point is made by patients with recurrent meningococcal infections, which can be linked to inherited deficiencies in terminal complement factors (Fijen et al. 1989; Lee et al. 1978; Owen et al. 2015). Whereas such individual genetic predispositions for particular infectious diseases are easily explained by defects in innate immune defence mechanisms, deletions or allelic variations in non-immune-related loci can also protect from infections as seen in biallelic loss-of-function (LOF) mutations of the fucosyltransferase 2 (*FUT2*) gene, which confer resistance to Noro- and Rotavirus infections (Lindesmith et al. 2003; Payne et al. 2015). In this context, it is becoming apparent that frequencies of such protective or deleterious alleles differ between human populations (Adrian et al. 2019), indicating that future G** × **E extrapolations should ideally consider human ancestry.

## Model refinement by G × E

Toxicology heavily depends on models (Lanzoni et al. 2019; Leist et al. 2008; Marx-Stoelting et al. 2021; Marx et al. 2020; Neuhaus et al. 2022; Pallocca and Leist 2022). Some models are so extensively used that it is at times forgotten that they are “only” modelling aspects of reality, but they are not the reality proper. This logical fallacy is called reification (Pallocca et al. 2022b), and one of its consequences has been an over-reliance on animal experiments in the past. Now, it has become clear that healing an “Alzheimer mouse” or protecting a rat from stroke or septic shock translates extremely poorly (less than 10%) to actual patients (Leist and Hartung 2013; Pistollato et al. 2016). The reasons for this discrepancy are not yet entirely clear, but genetic differences may play a role (Li et al. 2017; Seok et al. 2013). This assumption is in line with partially significant differences between the model systems. In some areas of toxicology, one animal species is 60% predictive of another one (Smirnova et al. 2018), and even strains of mice may show sensitivity differences to carcinogens by > 100-fold (Diwan et al. 1986; Romualdo et al. 2021). Understanding the underlying G** × **E effects is important for the selection of models with the best translational value in toxicology or in disease biology. Evidence for the importance of the G** × **E concept is provided by the absence of LPS-sensitivity in C3H/HeJ and C57BL/10ScCr mice. Their resistance to LPS-induced septic shock, compared to other inbred mouse strains, was the basis for the research leading to the 2011 Nobel Prize in Medicine (Poltorak et al. 1998).

Concerning cytotoxic properties, ouabain is an exemplary chemical that inhibits human Na/K-ATPase, but hardly affects the same enzyme in mice (Kent et al. 1987). The reproductive toxicity of thalidomide to New Zealand white rabbits, but not to other rabbit strains or mice, is another example (Knapp et al. 1962).

Also, the susceptibility of chicken to organophosphate-induced delayed-type neuropathy (relative to rodents) or the lack of effect of the neurotoxicant MPTP in rats (vs. the high sensitivity of some mouse strains and monkeys) represent well-established examples in toxicology. This suggests that knowledge of the relevant genetic backgrounds (including the gene products (proteins), and their regulation in models relative to human populations), would be highly beneficial (Li et al. 2017).

It is tempting to assume that all differences observed between animals and humans are due to genetic variation. Also, differences between animal strains (of the same species) are often considered to be fully explained by the different genomes. However, such simplistic assumptions neglect the large influences of life style, life history, husbandry and experimental protocol differences on the outcome of studies. Experiments repeated within a given animal model (same genes) can show a surprisingly large extent of variability. This characteristic may be due to animal husbandry, operator differences, or various lifestyle factors. Nevertheless, some of the variability is likely due to genetic heterogeneity, e.g. in outbred animal strains (Festing 2016). Various strategies have been suggested to control the genetic background and to use it for improved predictivity (Kafkafi et al. 2018; Richter et al. 2009; Vollert et al. 2022).

The key question of this editorial is: how can the G** × **E concept help to improve toxicological models and their predictions? The answer has two major, conceptually different, components. The first is obvious and has been frequently discussed: models may be selected to reflect optimally the human type of genetic background and the functional/regulatory networks encoded. For this reason, many human polymorphisms have been mapped to toxicant sensitivity differences (Axelrad et al. 2019; Chao et al. 2017; Gurol et al. 2023; Monte et al. 2022; Pham et al. 2022; Reverte et al. 2013; Salazar-Gonzalez et al. 2023; Xu et al. 2022a, b). If hazard evaluation follows the AOP concept, it is especially important that there are no large taxonomic differences and that the fine-tuning of key event relationships by genetic factors is similar between the model system and the human population. This aspect may require careful case studies and case-by-case evaluations, especially for complex networks (Arnesdotter et al. 2021; Knapen et al. 2020; Spinu et al. 2022).

The second issue is much less discussed. It deals with the uncertainty of predictions and with the population variability concerning toxic responses. In regulatory toxicology, and in risk assessment in general, not only is the determination of the insult level (toxicant dose or concentration) relevant, but also the uncertainty related to its measurement. The uncertainty describes the distribution of response thresholds across a population. If there is a narrow distribution, it is relatively simple to define a reference dose so that no individuals are exposed to toxic levels. If the variability is large, and possibly includes particularly sensitive subpopulations, it is much more difficult to define reference doses, and they may need to be set far lower than for the average population sensitivity. Genetic factors are a major contributor to the variability. Probabilistic risk assessment (Maertens et al. 2022) (see below) aims to take this inter-individual distribution into account.

Some promising studies have already been undertaken to understand genetic variability in model systems (Rusyn et al. 2022). Nevertheless, this research field is still in its infancy. Much research is required on defining—in principle—which factors are the main drivers of uncertainty and to which extent they can modify responses. If this field gets more advanced, many detailed case studies are required to see how such factors interact with various toxicants and in different models. In future, hopefully general rules and predictions can be derived from such data. To promote progress in this area, more awareness for G** × **E interactions and systematic testing in many models and with various insults is required. To highlight the need for a new view on G** × **E, we suggest some adaptations of naming, as detailed in the sections below. We anticipate that the work to provide a basis for quantitative predictions based on quantitative knowledge of complex biology may take decades. However, we feel that it is time to get it started (Leist et al. 2008), and to lay out a conceptual framework (Hartung et al. 2017; Mone et al. 2020; Pallocca et al. 2022a; Thomas et al. 2018, 2019).

## G × E and probabilistic risk assessment

The classical points-of-departure (PoD) for risk assessment are NOAELs. These are data points without any measure of variability, and with adversity determined by expert opinion, as opposed to stringent statistical criteria. The BMD concept is used increasingly, allowing for the calculation of a confidence interval. However, the statistical basis for this is usually weak, and the reasons for variation of the confidence intervals have not yet been sufficiently investigated. They are considered stochastic, rather than being causally driven by genetic differences in susceptibility. Usually, the PoD point estimates are divided by a safety factor (e.g. 100x) in a relatively rigid and formalized procedure to arrive at the reference dose (Dankovic et al. 2015). What could a future procedure look like? Knowledge and understanding of population variability may allow modelling of probability functions for toxicant risk at given exposure levels (Maertens et al. 2022). Regulatory toxicology could then use these functions to set reference doses so that 95, 99, or 99.99% of the population are protected (dependent on other factors and background knowledge used for risk management). In figurative terms, risk would be represented by a distribution curve, which is sliced in an appropriate (statistically defined) manner. This would also work the other way around: we could illustrate what the impact of different thresholds for public health are when deciding on such thresholds. This process may be combined with probabilistic uncertainty determinations within the test model. Instead of a point estimate, the PoD may be defined by the dose or concentration with a residual uncertainty of < 5% or < 1%.

## Broadened concept of G × E: G_*e *_× E_*t*_

To further develop the concept of G** × **E and set it on a solid basis (giving it an explanatory and quantitative character beyond non-mechanistic observations and qualitative descriptions), it is important to rationalize what is meant by “G” in the G** × **E expression. If we consider the protein-coding genes, it is the fluctuation in the level of proteins that determines cell behaviour. If a gene has a mutation, misses an exon, or is duplicated, this will, in most cases, have no direct (in a chemical sense) effect on cell behaviour and toxicant sensitivity. If the gene codes for an enzyme, its activity (*V*_max_, *K*_m_ and amount) is the key modifying factor. The same applies (with modifications) to transporters or transcription factors encoded by the gene. This statement, apparently trivial, has important implications. First, gene sequence changes outside the protein-coding region can play important roles, as they may affect the amount of protein produced. This aspect is also obviously extended to regulatory regions of specific genes, promoters and enhancers, whose mutations/polymorphisms can affect protein production. Second, the factor “G” can, in many/most cases, not be modelled with a binary approach (on/off or present/deleted). For many genes, dozens, hundreds or even thousands of mutations are known, so the activity of the corresponding proteins may vary nearly continuously over a relatively wide range. Third, epigenetic changes and other influences that affect the relevant mRNA levels must be taken into account, considering that it is the gene product that matters, not the polynucleotide as such. A hitherto often neglected genetic effect relevant to toxicity involves non-coding areas of the genome, including microRNAs, long non-coding RNAs, retrotransposon elements and satellite RNAs (Smirnova et al. 2012). An adversity arising independent of proteins is based on the hybridization of different nucleic acid species with one another, which can lead to R-loop formation, stalling of replication forks and generation of genetic instability (Janssen et al. 2018; Panatta et al. 2022a, b).

The role of gene expression in response to environmental changes (toxicant exposure, lifestyle, etc.) (vs the primary gene sequence) becomes evident when one considers the drastic sensitivity differences of various cell types in the body, or the influence that life stage and lifestyle can play in the toxicant response of one given individual. In both cases, the genes (primary nucleotide sequence) are unchanged (the same in all cells at all times). However, epigenetic modifications in each cell lead to the silencing of gene subsets or their positioning in open chromatin areas. This mechanism determines the subset of genes expressed in tissue-specific cell types. In addition, many other factors affect the mRNA levels of active genes, and eventually their translation. For this reason, “G” stands, in a functional sense, not only for the gene’s primary sequence, but also epigenetic state and transcript/protein levels. As “gene” is more narrowly defined in biological ontologies, it would cause less confusion to use a different acronym. We suggest here G_*e*_ (the subscript including epigenetics, expression levels and other relevant biology beyond the primary nucleotide sequence). This should signify a difference from the concept “gene” (unit of hereditary transmission), and be better suited to describe the influence of the genetic background in toxicology (Fig. 1). We would like to stress here, that epigenetic changes and transcriptome modifications are not necessarily correlated and thus need to be considered separately. Together they may contribute to the heritability of environmental influences, independent of the primary gene sequence alteration (Li et al. 2022; Siwek et al. 2020). N.B.: the concept does not neglect that it is mostly the concentration, localization and modification of proteins coded by the genes that determines the phenotype of cells. However, the role of non-coding RNAs and of long-range chromatin re-arrangements are also well noted (see outlook), but for space reasons not considered here.

## Direct effects of the environment and insult timing on G_***e***_

As described above, we have seen that considerations of the interaction between the “environment (E)”, i.e. the exposure situation to toxicants, and the genetic background are likely to lead to a better description and quantification of uncertainties in risk assessment than a focus on insult levels (exposures) alone. Toxicology is not the only field where this principle may be applied. It may be generalized to any harmful effects on humans, including natural ageing and long-term consequences of chronic stress. In all these cases, a name change from G to G_*e*_ may lead to a heightened awareness for the role that the affected biological system plays in the outcome of toxicant exposure.

There is a need for further adaptation of the concept: time must be given specific consideration. The duration of exposure is an important toxicological parameter, as expressed e.g. in Haber’s law (Macko et al. 2021) or in different testing methods for acute and chronic toxicity. We are well aware that time is part of the definition of E (long- or short-term exposures) in classical toxicology. However, many time concepts are still little considered, and awareness for them is important for new toxicological approaches. They include the time period of exposure during a sequence of life stages, the time between different exposures, the time of recovery after an exposure, etc. Time is also important as we apply the AOP framework. However, the AOP framework neither includes a symbol for time, nor is time a prominent descriptor of most key events or molecular initiating events. In addition, the development of qAOPs has seldom included time. There may be many reasons for not including time in qAOPs, but the paucity of data and absence of a time concept from the underlying AOP are key. Therefore, we suggest accentuating “E” with an extension for Time (E_*t*_) to highlight the need to take the various time-related issues into consideration.

The classical G** × **E concept regards environment as a neutral (positive or negative) driver of behaviour. For use in risk assessment, the term E_*t*_ should be considered to mainly stand for an insult (with its connotation of possibly detrimental effects). In summary, we suggest to rename G** × **E to G_*e*_** × **E_*t*_. E_*t*_ will mainly be considered as “insult” when exposure to xenobiotics is considered, but additional factors are not excluded (see below).

The consideration of G_*e*_ and E_*t*_ (both as orthogonal input factors) implies that they affect one another on a third dimension and thus together drive the outcome. However, in many cases, E_*t*_ will also directly affect *G*_*e*_. This concept can be investigated with a simple thought experiment: prolonged exposure may be perceived as a repetition of short exposures. After the first of these short exposure periods, G_*e*_** × **E_*t*_ needs to be considered. For instance, biological systems react to stressful stimuli by adaptations, i.e. the activation of canonical stress responses, such as the heat shock response, the oxidative stress response, the endoplasmic reticulum stress response, the DNA damage stress response, the inflammatory response, the hypoxia response, endotoxin tolerance etc. (Lehner and Hartung 2002; Ter Braak et al. 2022; Wink et al. 2018). All these canonical stress responses are adaptations of the transcriptional activities and epigenetic regulations of the cell. Consequently, after the first short exposure to an insult, the biological system would change its gene expression (G_*e*_) (Fig. 1C). This means that the second insult (or the prolonged insult period) would interact with a system that is altered concerning its G_*e*_-state. Thus, G_*e*_** × **E_*t*_ would be different from the first exposure period.

The above example shows that more prolonged insults will likely affect the G_*e*_ state of the system directly. The initial G_*e*_** × **E_*t*_ interaction will be different from that at the end of the exposure. One of the most extreme results of the time effect is ageing. Biological ageing may be measured by progress on an epigenetic clock (Li et al. 2022), i.e. a measureable alteration of the epigenetic state (G_*e*_) over time. This assumption means that the system changes its G_*e*_-state over time. Notably, the change of the G_*e*_ state over time does not parallel the progress of physical time. In other words, the biological clocks of individuals may run at a different speeds and humans of same “calendaric” age may have different biological ages (G_*e*_ states).

The system (e.g. human tissues) changes its G_*e*_ state because of environmental influences (E_*t*_), such as oxidative stress, DNA damage, etc. Exposure to DNA damaging conditions clearly changes the epigenetic state and, thus, the time of exposure affects G_*e*_ (Yang et al. 2023). In essence, this means that the environment (E_*t*_) can directly change G_*e*_ so that G_*e*_ and E_*t*_ are not really orthogonal dimensions that interact on a third level, but they also have some direct overlaps. The interaction of E_*t*_ and G_*e*_ thus occurs on two levels (direct and indirect), unless insults are of very short duration. In many cases (repeated/prolonged exposure), the system adapts, and the interaction of G_*e*_ and E_*t*_ becomes very dynamic.

This widened G_*e*_** × **E_*t*_ concept now allows for the incorporation of multiple environmental factors, acting sequentially or simultaneously. In classical toxicology, the latter case would be termed mixture toxicity. The concept would also allow for the interaction of toxicants, pathogens and/or lifestyle changes, with genetic backgrounds (Hartung 2022). For practical reasons, non-adverse environmental factors may even be considered as a third dimension.

With further development of such a concept, techniques to quantify G_*e*_** × **E_*t*_ (on different environmental backgrounds) may be identified and developed. The epigenetic clock (integrating the impact of multiple environmental factors over time with a given genetic background) may provide an initial orientation. Possibly, a set of epigenetic modifications and expression patterns of certain mRNAs may be defined for given cell types or toxicological situations to quantify the G_*e*_** × **E_*t*_ effect.

The integration of multiple environmental factors has already been explored in the field of mental illnesses and psychological distress, e.g. by combining knowledge of genetic variants of the serotonin transporter, early childhood experiences, and later life stress factors (Grabe et al. 2012). The research field dealing with chronic stress developed early on far-reaching concepts for how environmental exposure modifies the experimental system. This “allostatic load concept” (McEwen 1998) also integrated multiple inputs (e.g. direct stressors together with enhancing and moderating life style factors) to a fully developed model of what we named here G_*e*_** × **E_*t*_. The system alterations were read out in many ways, e.g. by a reduced telomere length upon continued stress (Epel et al. 2004) or altered inflammatory responses (Wirtz et al. 2003). In the course of these studies, it was revealed that pre-challenged systems (higher allostatic load, less resilience) would respond differently to a second or continued stressor, e.g. by accelerated ageing, coronary heart disease or altered brain function as consequence of an unbalanced hypothalamic–pituitary–adrenal (HPA) axis (Wirtz and von Kanel 2017). The implication of this is not only relevant to life-style-related stress. Exposure to toxic chemicals is a classical way to activate the physiological stress responses, and this plays a role in many toxicological studies (Everds et al. 2013).

With the increasing importance of non-animal methods in toxicology (Mone et al. 2020; Pallocca and Leist 2022), several questions arise concerning the G_*e*_** × **E_*t*_ concept in NAM. The answers are relevant to understand whether the novel toxicological test approaches will eventually allow predictions of human effects, not only concerning hazard of chemicals, but also concerning uncertainties of risk assessments. Here, brief information and food-for-thought on future research efforts is given.

## Can G_*e*_ × E_*t*_ be observed in NAM?

As many NAM are set up in a way to allow mechanistic studies and the application of modern ~ omics technologies, they seem pre-destined for studies on G_*e*_** × **E_*t*_ and underlying mechanisms. Many observational studies give evidence of this potential, while at the same time providing alerts concerning genetic variability. For instance, we (Kleensang et al. 2016) observed major differences in the transcriptional response to oestrogen in MCF-7 cells from different frozen vials. Similarly, (Gutbier et al. 2018a) correlated the toxicant sensitivity of LUHMES neurons with their culture history and corresponding development of mutational patterns. Such findings seem to be more widespread than assumed, as, e.g. (Ben-David et al. 2018) showed more broadly that cancer cell line drug responses continuously change by transcriptional evolution.

Other types of studies have made use of controlled shifts of the G_*e*_ status of cells. For instance, (Hammour et al. 2022) have compared two protocols to generate HepaRG cell models for drug toxicity studies, i.e. cells with the same DNA primary sequence were manipulated to assume different G_*e*_ states, then used further for G_*e*_** × **E_*t*_ studies. This approach is extremely widespread in the field of stem cell-derived cell types. A toxicological example is the use of one given iPSC line to generate two cell types (neurons and cardiomyocytes) that then showed different toxicological responses and were characterized by their transcriptome shifts (Seidel et al. 2022).

## Can one use NAM to evaluate and to predict the importance of G_*e*_ × E_*t*_?

Many different aspects have been addressed here, as exemplified by the small field of developmental neurotoxicity (DNT) testing. For instance, we (Modafferi et al. 2021) demonstrated that iPSC cells with or without an autism-related mutation in the CHD8 gene showed different neuronal development patterns. More importantly, evidence was obtained from this in vitro model that the DNT toxicant chlorpyrifos synergizes with the effect of the CHD8 variant. It has also been found that neuronally differentiating stem cells change their epigenetic structure upon toxicant exposure. This chromatin modification affected gene expression patterns at later developmental stages (Balmer et al. 2014). Using a similar experimental model, a genome-wide prediction approach was developed on how toxicant exposure would affect gene expression patterns over time (Meisig et al. 2020).

NAMs also allow interventions on selected candidate genes, or they can be used for genome-wide assessments to identify susceptibility genomic loci. Genetic screens based on CRISPR and shRNA libraries have been used to identify specific genes of interest associated to altered susceptibility to toxicants: (Olivieri et al. 2020) generated, with a massive effort of 31 genome-wide screens against 27 genotoxic agents, a genetic map of human cell responses to DNA damage inducers.

When model systems are used, considerations of the environment are particularly important, as they are decided, defined and maintained by the experimenter, often independent of G_*e*_ and independent of E_*t*_. In a *narrow G*_*e*_ ×  E_*t*_* concept*, all environmental factors may be included in “E_*t*_”. In a wider concept, a third dimension may be justified. Often overall G_*e*_** × **E_*t*_ effects depend on the cell density, on the cell culture medium, on the oxygen supply of cells (Pamies and Hartung 2017), and on other factors that neither affect gene expression, nor can be classified as insults in a classical sense. A striking example is neurotoxicity testing, where the contents of glucose and galactose in the medium can affect toxicant potency by > 1000-fold (Delp et al. 2019). Media composition can also cause drastic shifts in cell death type or extent (Gutbier et al. 2020; Leist et al. 1997; Suciu et al. 2023).

In applied toxicology, it is a common approach to focus on the insult effect (toxicant exposure) of the environment. This approach neglects the contributions of all other aspects of the environment. For a comprehensive risk assessment, it appears mandatory to also take the beneficial effects of the environment into account. This extension is an important background frame for the G_*e*_** × **E_*t*_ concept, and also points to needs for future adaptations.

## Can one explore the human population G_*e*_ × E_*t*_ in NAM?

In parallel to the exemplary studies above, generalized approaches have been developed on how the genetic component of G_*e*_** × **E_*t*_ may be decoded using NAM approaches. Such approaches are widespread in oncology, and a more modern variant is the generation of patient-derived cancer organoids for drug screening (Driehuis et al. 2020). For instance, the NCI-60 cancer cell line panel has been used to study the correlation of toxicant effects with genetic features of the cells (Krushkal et al. 2021). This experimental approach will generate large data sets that will eventually allow the correlation of drug toxicities with genetic backgrounds and the deconvolution to susceptibility and sensitizing genetic modules. Another approach was developed on the basis of immortalized human lymphoblastoid cell lines (Abdo et al. 2015a; O'Shea et al. 2011). In this approach, e.g. 1086 cell lines from the 1000 Genomes Project were used to assess variation in cytotoxic response to 179 chemicals (Abdo et al. 2015a, b). Similar approaches are ongoing with human iPSC or primary human hepatocytes, and sequencing of the cells will allow multivariate correlations between genetic backgrounds and toxicant susceptibilities.

An outlook towards the future is provided by single cell sequencing approaches, where the response of individual cells in complex multi-cellular cultures can be characterized, and where cells with different genetic background can be combined in a single culture system. The technology is being made ready for toxicological applications (Liu et al. 2022; Paisley and Liu 2021). Single-cell sequencing was applied to simple liver models already years ago (Zhang et al. 2020). More recently, an exemplary study on a 3D microtissue liver model revealed cell type-specific responses to pre-fibrotic stimulation (Messner et al. 2021). An increase of complexity is provided by a microfluidics-based stem cell model of early post-implantation human development for teratology studies. It allows following the toxicant effects on single cells by RNA sequencing (Zheng et al. 2021).

On the basis of such studies, one vision for the future is that toxicant testing will be performed with assays that use multiple test systems representative of the human genetic variability (Fig. 1D, E). This means that the future test methods will not be based on a single cell line, cultured under highly defined conditions, as is currently often the case. Instead, cells with multiple genetic backgrounds and also in various transcriptional and epigenetic states may be used in a highly parallelized way to fully explore the extent of uncertainty linked to the model system variation.

## Conclusions and outlook

The G** × **E concept is neither new, nor has it gone unnoticed. It is firmly established in toxicology and other disciplines. So why is it worth revisiting and investing thoughts and resources? Three arguments have been brought forward, and are summarized here:

First, the limitation of “G” to a gene primary sequence is too restrictive, and the concept that a gene is either on or off is outdated. The G_*e*_-concept, referring to transcriptome adaptations, non-coding RNAs, epigenetics and various factors affecting the encoded proteins appears more appropriate. This has not only conceptual consequences, but also affects the parameters that need to be assessed and the techniques used for this.

Second, the “E” component should be considered broadly, and it needs to be considered that it may feedback on G_*e*_. The timing of exposure is a key issue often neglected, and the interaction of multiple insults or of insults with other life style factors is a frequent difference between simple model systems and the real world of human life. Where models are being used for predictions of human health, this has important consequences. In an integrated view, a step for the future would not only be to apply the concept to toxicology, infection research, stress-fatigue etc., but to see these fields as part of an integrated structure, as illustrated by an exemplary study on the interaction of viral infection, immune modulation by helminths and air pollution (Leist et al. 2014).

Third, while the concept is broadly embraced, there is a long way to go from a conceptual understanding to a quantitative application. Initially, a mapping of the key drivers is required. For each of them, it needs to be determined, how much it can alter the adverse outcome. This would already in some cases allow a more rational definition of uncertainty of toxicological predictions. Finally, a network model for all drivers of variability and their interaction has to be developed, based on systems biology understanding. This is a long-term vision, possibly taking decades. The effort would pay not only for some practical applications, but also by putting disease biology on a new foundation. For the whole field of medicine/biology, it may be debated whether this is an ambitious, but realistic goal, or an unachievable dream. However, partial solutions may be developed for more narrow fields and applications. For instance, the concept of AOP in toxicology is taking the step towards quantitative AOP (mathematical networks predicting the state of the system and its components over time). In parallel, the mostly linear AOPs are converted to AOP networks. First approaches to determine uncertainties for individual key events in such networks have been started (Paini et al. 2022; Spinu et al. 2022; Tebby et al. 2022; Yang et al. 2022). Application of the G_*e*_ × E_*t*_ concept to key event definitions may be an important next step. Concerning research policies and research funding, it appears vital to develop a roadmap for such a process, similar to other long-term initiatives, such as the transition from animal-based testing to NAM (Leist et al. 2014). Steps to move towards integrated biomarkers (e.g. multi-omics approaches or exploring the use of exosomes as integrative biomarkers) may be integrated (Cano et al. 2023; Gupta et al. 2021; Jain et al. 2023; Perpetuo et al. 2022; Pitzer et al. 2023).

## Abbreviations

ADI: Acceptable daily intake
ADME: Absorption-distribution-metabolism-excretion, aka toxicokinetics (TK)
AOP: Adverse outcome pathway
BMD: Benchmark dose
*C*_max_: Peak concentration in plasma
E_*t*_: Timing of toxic exposures/insults
G_*e*_: Sequence, epigenetic state, and transcript levels of genes of an organism/model system and their translation to the proteome
G** × **E: Classical genes vs environment concept
G_*e*_** × **E_*t*_: Interaction of the biological (G_*e*_) background and environmental exposure, with emphasis on timing of toxic exposures/insults (E_*t*_)
LOEL: Lowest observed effect level
MAK: Maximal workplace concentration
NAM: New approach methodologies
NOAEL: No observed adverse effect level
NOEL: No observed effect level
PoD: Point-of-departure
RfD: Reference dose
SF: Safety factor, aka uncertainty factor
